# Synthesis of Linearly Fused Benzodipyrrole Based Organic Materials

**DOI:** 10.3390/molecules21060785

**Published:** 2016-06-17

**Authors:** Maarten Vlasselaer, Wim Dehaen

**Affiliations:** Molecular Design and Synthesis, Department of Chemistry, KU Leuven, Celestijnenlaan 200F, 3001 Heverlee, Belgium; maarten.vlasselaer@chem.kuleuven.be

**Keywords:** indolo[3,2-*b*]carbazole, organic electronics, pyrrolo[2,3-*f*]indole

## Abstract

The objective of this review is to give an overview of the synthetic methods to prepare different indolo[3,2-*b*]carbazoles and similar systems with a potential use in electro-optical devices such as OLEDs (organic light emitting diode), OPVs (organic photovoltaic) and OFETs (organic field effect transistor). Some further modifications to the core units and their implications for specific applications are also discussed.

## 1. Introduction

Polycyclic compounds containing two pyrrole rings have been widely studied because they possess many interesting properties. One of them is the good charge transfer properties these type of products possess [1,2], a second one is the feasibility to tune the electronic levels of these compounds for different applications. This causes these compounds to be excellent candidates for applications such as OPVs (organic photovoltaics) [3,4], DSSCs (dye-sensitized solar cell) [5], OLEDs (organic light emitting diodes) [6,7], and OFETs (organic field effect transistor, including thin film transistors) [8,9,10]. Advantages of organic materials for these applications are potentially low cost [11], lightweight and flexibility.

**Figure 1:**
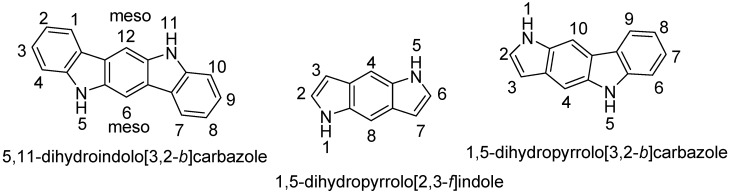
Structure of indolo[3,2-*b*]carbazole, pyrrolo[2,3-*f*]indole and pyrrolo[3,2-*b*]carbazole.

The main focus of this review will be the indolo[3,2-*b*]carbazoles, but also smaller benzodipyrrole systems like pyrrolo[2,3-*f*]indoles and pyrrolo[3,2-*b*]carbazoles (Figure 1), larger systems, and heterocyclic analogs of indolo[3,2-*b*]carbazole will be discussed. 

The smaller systems can be considered as indolo[3,2-*b*]carbazoles with one or two of the outer benzo-rings missing. The larger systems have one or more extra rings compared to indolo[3,2-*b*]carbazole. We will only focus on the linear indolo[3,2-*b*]carbazole isomer, and its smaller and larger analogs in this review due to the more interesting spectroscopic properties [12].

In this review, we will discuss the synthesis of the parent indolo[3,2-*b*]carbazole scaffold and further functionalization and polymerization of this compound for applications such as OPVs, OLEDs and OFETs. However, the focus of the review is synthetic and we will not go in the details of the applications.

**Figure 2:**
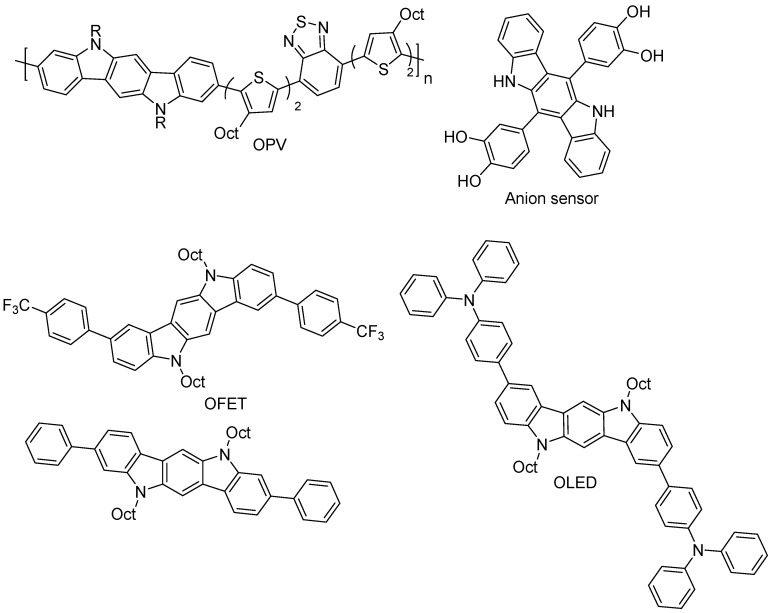
Some examples of indolo[3,2-*b*]carbazole and their applications.

Another interesting application is the use of indolo[3,2-*b*]carbazole as anion sensor in aqueous environment [13] and the biological activity of indolo[3,2-*b*]carbazole [14,15,16]. In Figure 2, some examples of indolo[3,2-*b*]carbazoles tested in the above-mentioned applications are given.

## 2. Indolo[3,2-*b*]carbazoles: Synthesis

### 2.1. Oxidative and Transition Metal Catalyzed Synthesis

**Scheme 1:**
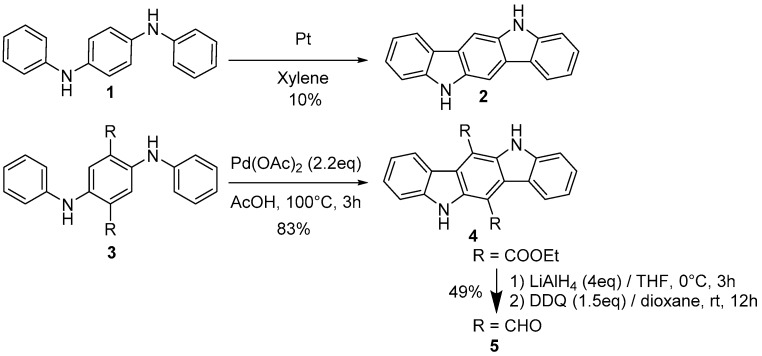
Cyclodehydrogenation of *N*,*N*′-diphenyl-*p*-phenylenediamine.

The first synthesis of indolo[3,2-*b*]carbazole was reported by Grotta *et al.* They used *N*,*N*′-diphenyl-*p*-phenylenediamine **1** and platinum to perform a cyclodehydrogenation to obtain unsubstituted indolo[3,2-*b*]carbazole **2** in 10% yield (Scheme 1) [17]. Lamm *et al.* performed the same reaction on the dimethylated precursor, which was closed photochemical to indolo[3,2-*b*]carbazole in 10% yield. The electrochemical properties of this material were investigated [18,19]. Chakrabarty *et al.* used a similar method, but they started from 3-aminocarbazole to perform one photochemical cyclization to obtain indolo[3,2-*b*]carbazole [20].

Bergman *et al.* used palladium acetate as an oxidizing agent to perform a similar ring closure to obtain indolo[3,2-*b*]carbazole **4** in much higher yield (83%) starting from disubstituted *N*,*N*′-diphenyl-*p-*phenylenediamine **3**. The ester groups on indolo[3,2-*b*]carbazole **4** were further converted to di-aldehyde **5** in very good yield (Scheme 1) [21].

**Scheme 2:**
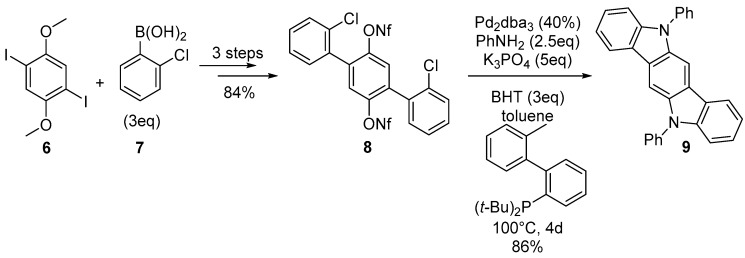
Pd-catalyzed quadruple *N*-arylation.

Nakano *et al.* started with 1,4-diiodo-2,5-methoxy-benzene **6** to perform a double Suzuki coupling with 2-chlorophenylboronic acid **7**. The obtained product was demethylated and converted to the nonaflate ester **8** in two steps. This compound underwent two double Buchwald–Hartwig aminations with aniline to ultimately give the indolo[3,2-*b*]carbazole **9**. It has also been proven possible to synthesize asymmetrical indolo[3,2-*b*]carbazoles by performing the Suzuki couplings in a stepwise manner (Scheme 2) [22].

Chang *et al.* developed a general oxidative method starting from *N*-substituted amidobiphenyls to prepare carbazoles. In order to obtain a high yield, electron withdrawing groups such as acetyl or phenylsulfonyl should be placed on the amines. Alkyl substituted analogs seem to be disfavorable for the reaction.

**Scheme 3:**
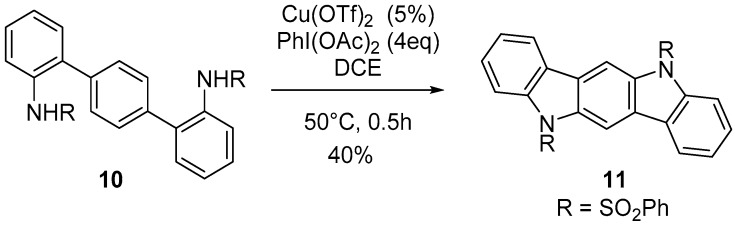
Oxidative copper catalyzed synthesis.

PhI(OAc)_2_ was shown to be the stoichiometric oxidant with the best results for the carbazole synthesis. Copper triflate was used as a catalyst and this improved the yield of the reaction going from 75% up to 93%. The optimized reaction conditions (for carbazole) were used on 2,2″-bis(sulfonamide)-*p*-terphenyl **10** to afford indolo[3,2-*b*]carbazole **11** in 40% yield after a double cyclization (Scheme 3) [23].

### 2.2. Synthesis Starting from Indoles

**Scheme 4:**
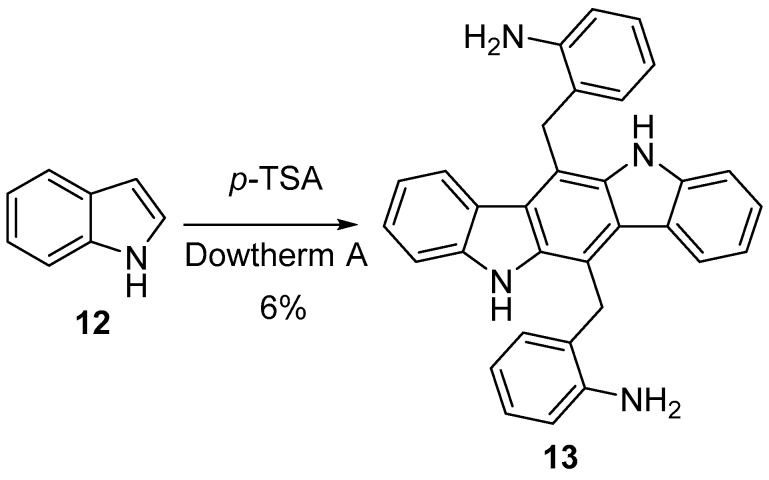
Oligomerization of indole.

Ishii *et al.* investigated the oligomerization of indole in acidic conditions. One of the compounds the authors found in the mixture obtained by combining indole **12** with *p*-toluenesulfonic acid in Dowtherm A (mixture of biphenyl (26.5%) and diphenyl ether (73.5%)) was indolo[3,2-*b*]carbazole **13**, but only 6% yield was obtained under these conditions (Scheme 4) [24]. The generality of this method was not investigated. Korolev *et al.* treated 3-formylindole with acid and they detected several indolocarbazoles [25].

**Scheme 5:**
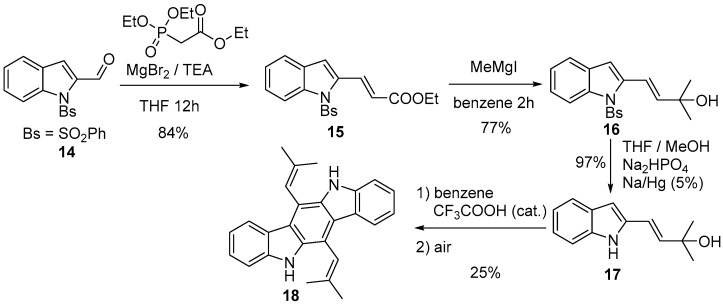
Dimerization of tertiary alcohol.

Cheng *et al.* started their synthesis from *N*-benzenesulfonylindole-2-carbaldehyde **14**, which was subjected to a Horner–Wadsworth–Emmons reaction to obtain the corresponding cinnamate ester **15**. This compound was then reacted with an excess of methylmagnesium iodide to obtain the tertiairy alcohol **16**. The protecting benzenesulfonyl group was removed and then the obtained compound **17** was treated with a catalytic amount of acid, which generated a stabilized cation that dimerized head-to-tail in acidic conditions. The resulting tetrahydro compound (structure not shown) was further oxidized with air oxygen to obtain the indolo[3,2-*b*]carbazole **18** (Scheme 5) [26].

**Scheme 6:**
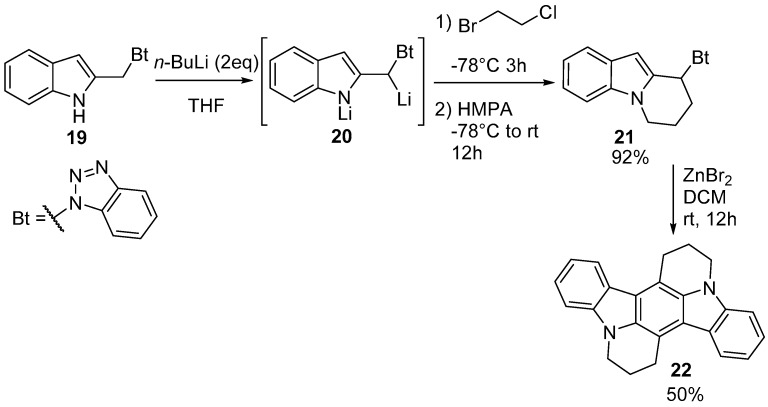
Katritzky indolocarbazole synthesis.

Katritzky *et al.* prepared indolo[3,2-*b*]carbazoles starting from 2-[(benzotriazol-1-yl)methyl]indole **19**, which was lithiated twice to form intermediate **20** and then *C*,*N*-dialkylated with 1-bromo-3-chloro-propane. The resulting tricyclic compound **21** was then converted using ZnBr_2_ as a catalyst into a cationic intermediate which dimerizes. The intermediate tetrahydroindolocarbazole (not shown) was oxidized with ambient oxygen to obtain the doubly fused indolo[3,2-*b*]carbazole **22** in 50% yield (Scheme 6) [27]. This synthesis is based on earlier work by Katritzky *et al.* in which benzotriazole was also used as a leaving group to perform a coupling reaction between indole and heteroaromatic structures. The overall yield of these reactions is however lower (22% starting from the corresponding benzotriazole compound) [28].

**Scheme 7:**
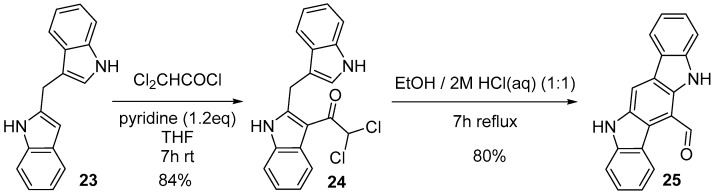
Synthesis of 6-formylindolo[3,2-*b*]carbazole from 2,3′-diindolylmethane.

Bergman *et al.* prepared 6-formyl-indolo[3,2-*b*]carbazole **25** starting from 2,3′-diindolylmethane **23**, which was prepared in several steps. This compound was then condensed with dichloroacetylchloride in THF using pyridine as a base (84%) to form acylated compound **24**. Acid catalyzed ring closure and hydrolysis of the dichloromethyl function at the meso position afforded the 6-formyl-indolo[3,2-*b*]carbazole **25** (80%) (Scheme 7) [29].

**Scheme 8:**
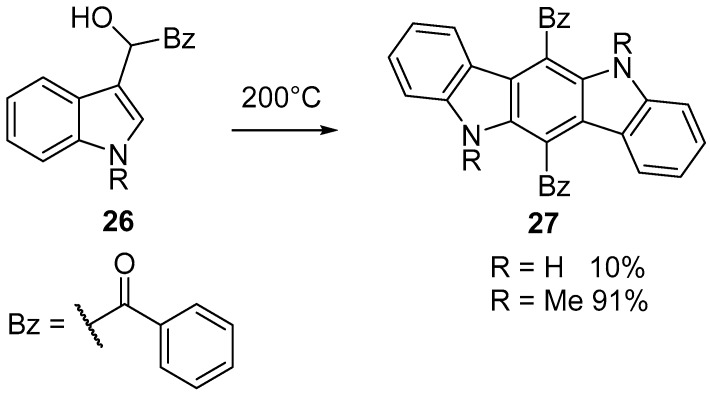
Thermal synthesis of indolo[3,2-*b*]carbazole from indole-3-carbinols.

Ivonin and coworkers used indole and phenylglyoxal to prepare 2-hydroxy-2-indol-3-yl-acetophenone **26**, which was then heated up to 200 °C. When the non-alkylated indole is used, only 10% of the dibenzoylated indolo[3,2-*b*]carbazole **27** is obtained. By using *N*-methylated indole for this reaction, the yield is increased up to 91% (Scheme 8) [30].

**Scheme 9:**
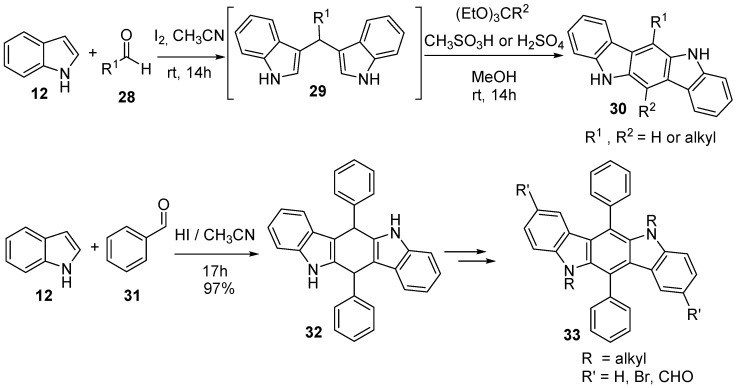
Condensation of indole with aldehydes.

Dehaen *et al.* developed a strategy to prepare indolo[3,2-*b*]carbazoles starting from 3,3′-diindolylmethane **29**. This diindolylmethane was obtained *in situ* by Bronsted- or Lewis acid-catalyzed condensation of indole **12** and an aliphatic aldehyde **28**. The weak Lewis acid iodine was used in this case. In the second step, an orthoester and a strong Bronsted acid have been used to perform the ring closure (20%–50%). Previous to the ring closure, the 3,3′-connected diindolylmethane rearranged to a 2,3′-connected isomer to ultimately give indolo[3,2-*b*]carbazole **30** (Scheme 9) [31].

Another related method is the direct condensation of indole **12** with benzaldehyde **31** in the presence of hydrogen iodide to obtain 6,12-diphenyl-5,6,11,12-tetrahydroindolo[3,2-*b*]carbazole **32** in excellent yield in a single step. This compound then can further be functionalized and converted to the fully aromatic indolo[3,2-*b*]carbazole **33** (Scheme 9) [32].

**Scheme 10:**
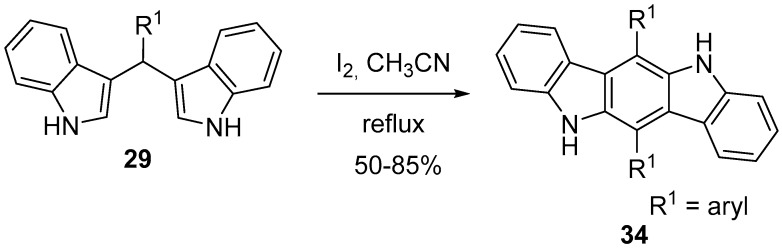
Dimerization of 3,3′-bisindolylmethanes.

Bhuyan *et al.* started their synthesis from isolated 3,3′-diindolylmethanes **29**. The starting material was dimerized with iodine as a catalyst to achieve a symmetrical indolo[3,2-*b*]carbazole **34** in 50%–85% yield after 35 min. One equivalent of indole was left unreacted after elimination from the starting material. The final compound is symmetrical because the use of orthoformates is not required. The reaction however does not work with aliphatic and strong electron withdrawing aromatic R groups (Scheme 10) [33]. Another method by Bhuyan *et al.* is the three component reaction of indole with an aldehyde and *N*,*N*-dimethylbarbituric acid, which affords a 3-alkylindole that can dimerize to a symmetrical indolo[3,2-*b*]carbazole [34].

Mohanakrishnan *et al.* developed a method where 2-methyl-indole-3-carboxaldehyde **35** has been used as a starting material. The aldehyde group was condensed with diethyl malonate and the 2-methyl group was brominated to obtain **36**. This biselectrophilic compound can then be condensed with various electon rich (hetero)aromatic systems under the influence of a Lewis acid. *N*-alkyl-indole **37** has thus been used to obtain indolo[3,2-*b*]carbazole **38** in 55% yield after elimination of diethyl malonate [35].

**Scheme 11:**
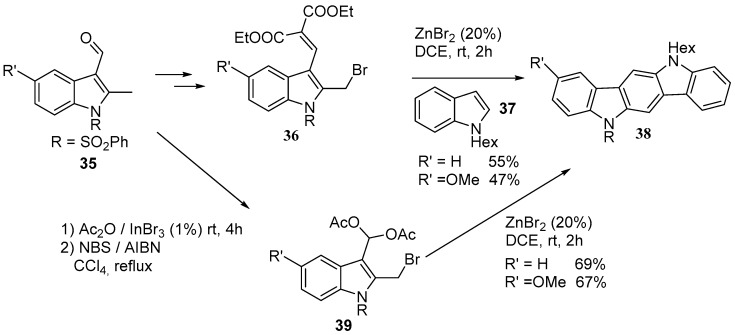
Lewis acid catalyzed condensation.

Later, the aldehyde was converted to an acetal as an alternative to the condensation with diethylmalonate. Again, the methyl group was brominated. The obtained bis-electrophile **39** can be condensed with various aryl- and heteroaryl rings to get a polycyclic system. When an *N*-alkyl-indole **37** was used, in combination with ZnBr_2_ as a Lewis acid catalyst, indolo[3,2-*b*]carbazole **38** was formed in 67%–69% yield. The bromide leaving group can also be replaced by an acetate [36]. The yield is however lower in this case (54%) (Scheme 11).

**Scheme 12:**
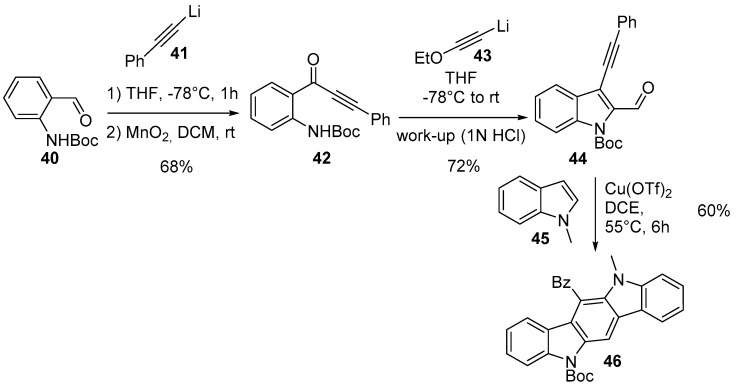
Condensation of indole with 3-alkynyl-indole-2-carboxaldehyde.

Reddy *et al.* prepared funtionalized indoles starting from *N*-Boc protected 2-aminobenzaldehyde **40**. Nucleophilic attack of lithiated alkyne **41** and successive oxidation gave compound **42**, which was converted by combination with 1-lithio-2-ethoxyethyne **43**, acidic deprotection and cyclization to 3-alkynylindole-2-carboxaldehyde **44**. This compound was then condensed with 1-methyl-indole **45** in oxidative conditions, using copper(II)triflate, to obtain indolo[3,2-*b*]carbazole **46** in 60% yield (Scheme 12) [37].

### 2.3. Fischer Indole Synthesis

**Scheme 13:**
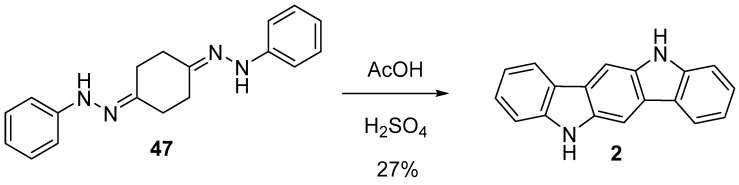
Fischer indolo[3,2-*b*]carbazole synthesis.

Robinson was the first to prepare the indolo[3,2-*b*]carbazole scaffold by performing a double Fischer indolization. He started from bishydrazone **47** to obtain the indolo[3,2-*b*]carbazole **2** in 27% yield, using a mixture of sulfuric acid and acetic acid (Scheme 13) [38]. 

**Scheme 14:**
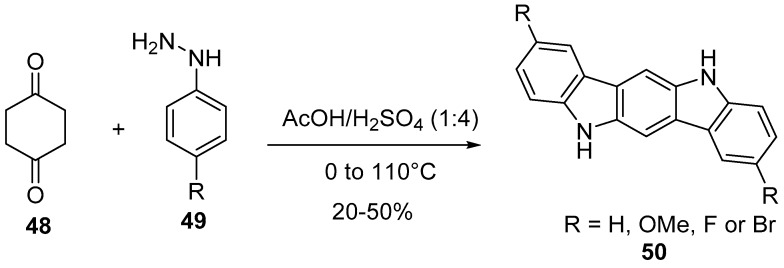
Fischer indolo[3,2-*b*]carbazole synthesis.

Bergman *et al.* also exploited the Fischer indole synthesis to prepare functionalized indolo[3,2-*b*]carbazoles starting from 1,4-cyclohexanedione **48** and functionalized phenylhydrazines **49** (Scheme 14). The indolo[3,2-*b*]carbazoles **50** were prepared in 20%–50% yield, which is an improvement compared to the other synthesis described in the same paper using functionalized arylamines and Pd(OAc)_2_ (10%) [39]. See Ong *et al.* for extra examples [40].

### 2.4. Cadogan Synthesis

**Scheme 15:**
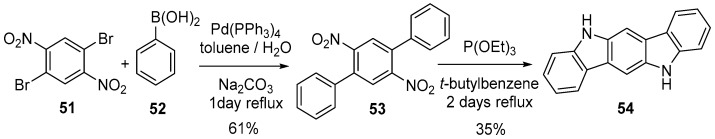
Double Cadogan ring closure.

Müllen *et al.* were the first to prepare indolo[3,2-*b*]carbazole via a Cadogan ring closure. First, they prepared terphenyl compound **53** by performing a double Suzuki coupling on 1,4-dibromo-2,5-dinitrobenzene **51** and phenylboronic acid **52** in 61% yield. Then, the double Cadogan ring closure was performed to obtain the final compound **54** (Scheme 15) [41]. For another example see Leclerc *et al.* [42].

**Scheme 16:**
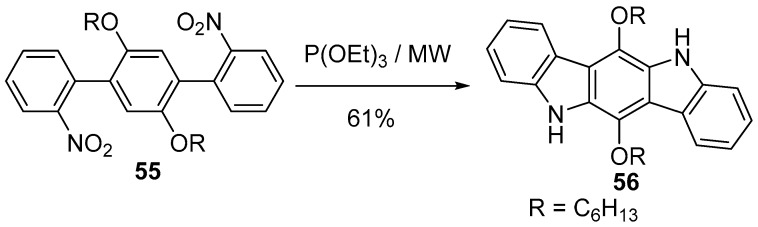
6,12-dialkoxy-indolo[3,2-*b*]carbazole.

Wrobel *et al.* exploited a microwave assisted Cadogan ring closure on terphenyl **55** to obtain 6,12-dialkoxy-indolo[3,2-*b*]carbazole **56** in reasonable yield (Scheme 16) [43].

### 2.5. Oxidation of Indolo[3,2-b]carbazole

**Scheme 17:**
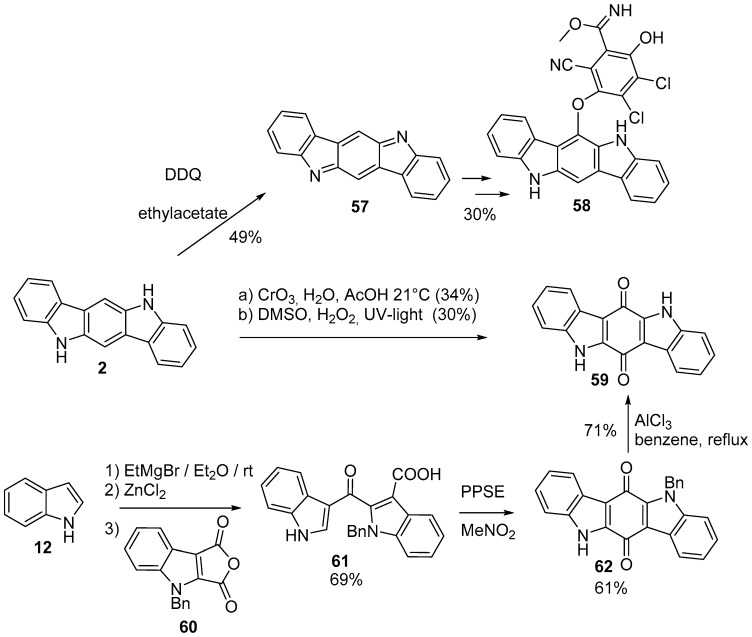
Synthesis of oxidized indolo[3,2-*b*]carbazole.

5,11-Dihydro-indolo[3,2-*b*]carbazole **2** can be oxidized to indolo[3,2-*b*]carbazole **57**. This compound however is reactive towards nucleophiles and the reduction product of DDQ will do an addition on the oxidized indolo[3,2-*b*]carbazole to obtain the meso substituted compound **58** (Scheme 17) [44]. By putting *t*-butyl groups on the structure of **57**, the oxidized molecule is stable and could be isolated and characterized by X-ray crystallography [45].

The Bergman group prepared the indolo[3,2-*b*]carbazole-6,12-dione **59** in fair yield by oxidizing indolo[3,2-*b*]carbazole **2** at the *meso*-positions with CrO_3_ (34%) or H_2_O_2_ (30%).

The same compound **59** can be obtained by reaction of anhydride **60** with metallated indole **12**, followed by acid-catalyzed ring closure of the bisindole ketoacid **61** in a polar solvent and deprotection of **62** (30% overall yield) (Scheme 17) [46]. Substituted derivates of **59** were prepared by Youssef *et al.* by reacting substituted anilines and tetrabromo-*p-*benzoquinone in a three step reaction. Also similar ring expanded systems were prepared by this method [47].

## 3. Indolo[3,2-*b*]carbazoles: Functionalization and Polymerization 

**Scheme 18:**
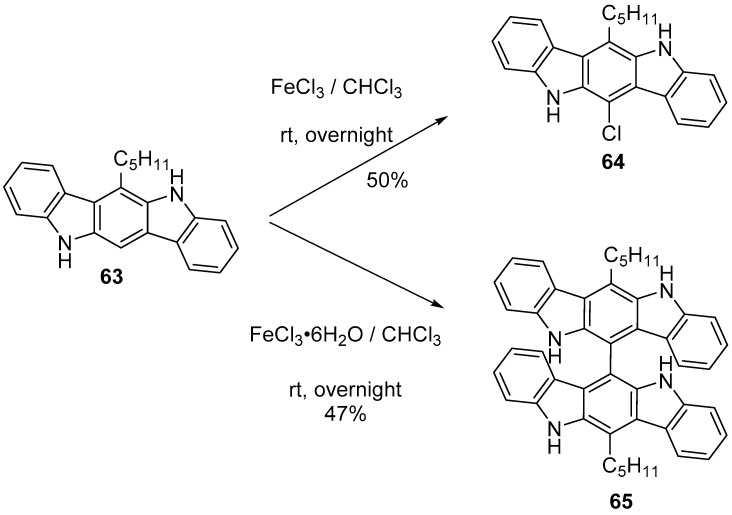
Chlorination and dimerization of indolo[3,2-*b*]carbazole.

Dehaen *et al.* functionalized non-alkylated indolo[3,2-*b*]carbazole **63** using FeCl_3_. When using anhydrous FeCl_3_, indolo[3,2-*b*]carbazole **63** was chlorinated at the 12 position to obtain **64**. Another objective was to form dimer **65**, which was also detected in the previous reaction (<5%). When the hydrated form, FeCl_3_•H_2_O, was used, dimer **65** was formed in 47% yield (Scheme 18) [48].

The indolo[3,2-*b*]carbazoles **63** obtained by Dehaen *et al.* with a free *meso*-position can be *N*-alkylated (83%) or -arylated (53%–70%) twice to obtain **66**. Sulfonation only occurs one time, at the nitrogen next to the free meso-position (60%). The free nitrogen can then be arylated in 70% yield to form indolo[3,2-*b*]carbazole **67** [48].

A similar double *N* arylation was also performed by Hu *et al.* using 1-iodonaphtalene and substituted iodobenzenes, using even more drastic conditions [49,50].

**Scheme 19:**
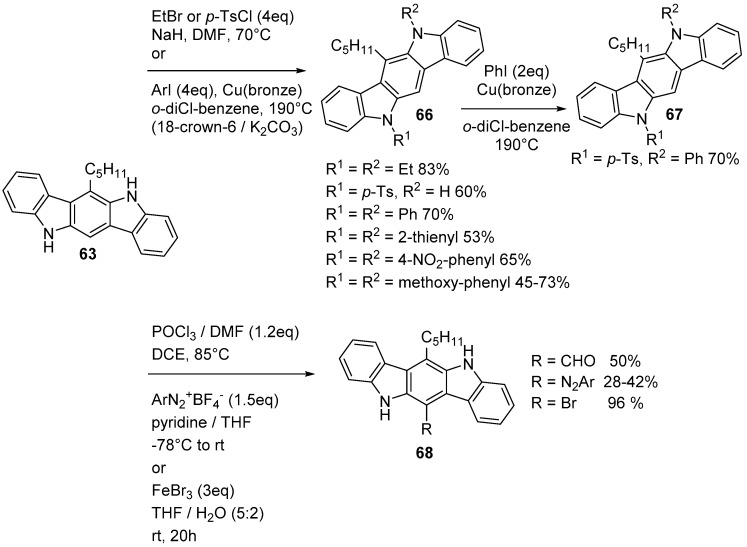
Functionalization of indolo[3,2-*b*]carbazole.

When using non-alkylated indolo[3,2-*b*]carbazole **63**, the free meso-position has been found to be the most reactive one for formylation (50%), bromination (96%), and diazotation (28%–42%) to get indolo[3,2-*b*]carbazole **68** (Scheme 19) [31,51]. Brominated compound **68** was alkylated and then converted to 6-(4′-formylphenyl)-5,11-dimethyl-12-pentyl-indolo[3,2-*b*]carbazole, which was then used by Maes *et al.* to prepare meso-substituted porphyrins [52].

**Scheme 20:**
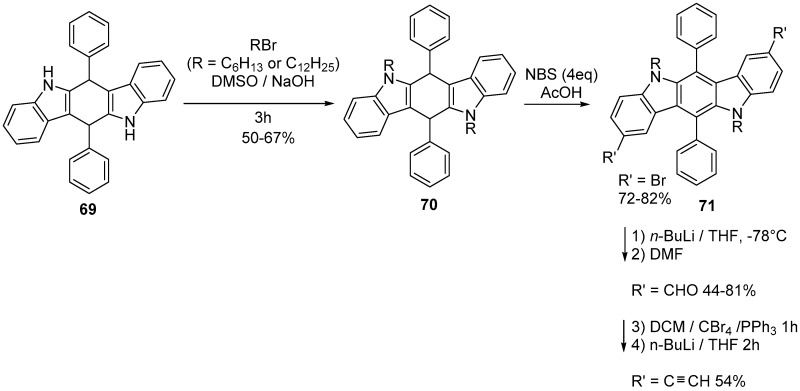
Functionalization of indolo[3,2-*b*]carbazole.

The tetrahydroindolocarbazole **69** which was obtained when benzaldehyde and indole were used for the condensation, has phenyl groups as substituents at both meso position. The compound is however not yet fully aromatic. The tetrahydroindolo[3,2-*b*]carbazole is first alkylated twice in 50%–67% yield to the more soluble indolo[3,2-*b*]carbazole **70** and then brominated with an excess of NBS, which at the same time aromatizes the middle ring, to obtain dihydroindolocarbazole **71**. These bromine atoms can be converted to aldehydes and further to alkynes (Scheme 20) [32].

Grazulevicius *et al.* prepared indolo[3,2-*b*]carbazole polymers to be used as hole transporting materials and as emitting layer in OLEDs. The active material was prepared by alkylation of nitrogen and Buchwald–Hartwig amination of dibrominated indolo[3,2-*b*]carbazole **72** to prepare indolo[3,2-*b*]carbazole **73** which then was polymerized with acid catalysts. 

Indolo[3,2-*b*]carbazole **74** is functionalized at the free meso position with hydrazones to form **75**.

The alkyl and hydrazon functionalities contain reactive groups like oxetanes or vinyl groups.

**Scheme 21:**
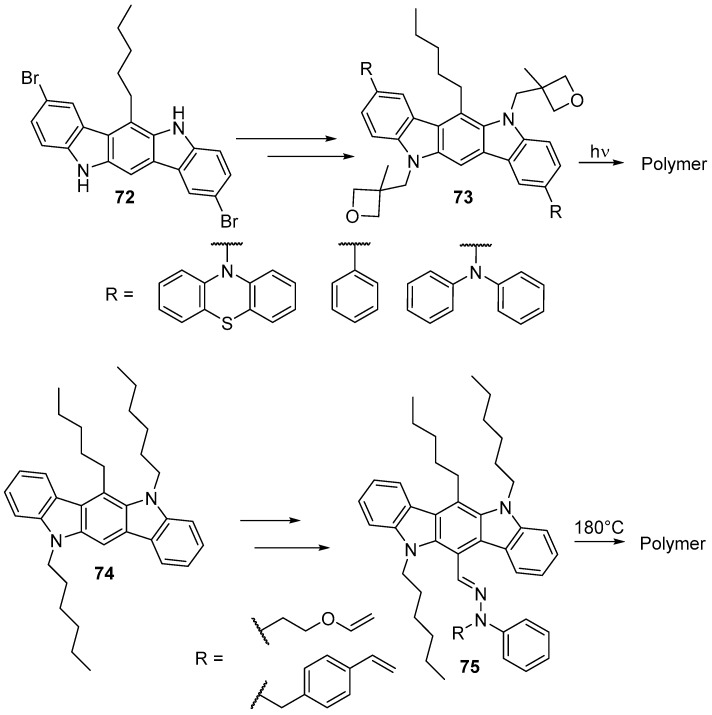
Synthesis of cross linkable indolo[3,2-*b*]carbazoles.

These are introduced in the molecules to enable self-polymerization at high temperatures (up to 180 °C) to obtain a more stable morphology (Scheme 21) [53,54]. Also epoxides were used to crosslink indolo[3,2-*b*]carbazoles under influence of aromatic dithiols [55]. A similar polymerization reaction with vinyl end-capped indolo[3,2-*b*]carbazoles was also performed by Nagase *et al.* after alkylation of one nitrogen atom of the indolo[3,2-*b*]carbazole scaffold [56]. Jiang *et al.* hypercrosslinked indolo[3,2-*b*]carbazoles using FeCl_3_ as a catalyst and dimethylformamide dimethylacetal as the crosslinker [57].

Irgashev *et al.* developed methods to introduce formyl and acyl groups on the 2- and 8-position of indolo[3,2-*b*]carbazole **76**. The best results for the formylation reaction were obtained by using the “Rieche method”, using SnCl_4_ and dichloromethylpentyl ether in excess. The di-formylated compound **77** was obtained in 80% yield [58].

**Scheme 22:**
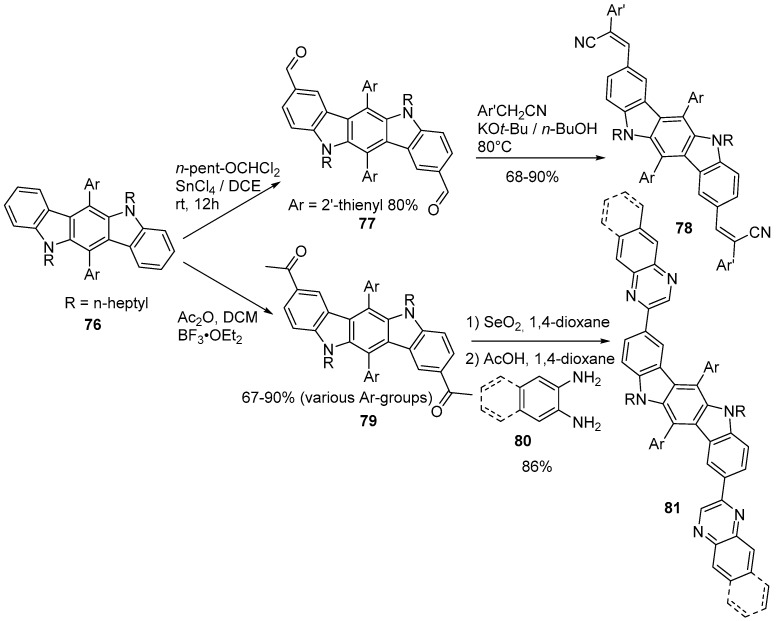
Formylation and acetylation of indolo[3,2-*b*]carbazole.

Diacetylation of indolo[3,2-*b*]carbazole **76** was performed in 67%–90% yield, using BF_3_•OEt_2_ to obtain indolo[3,2-*b*]carbazole **79** [59]. The prepared aldehydes and acetyl groups were further used to couple indolo[3,2-*b*]carbazole with various electron withdrawing groups to get donor–acceptor systems **78** and **81** (Scheme 22). Compounds **78** showed a red shift in the absorption spectrum (onset around 470–550 nm) compared to the parent indolo[3,2-*b*]carbazole **76** (onset around 430–440 nm with low absorption). The (benzo[g])quinoxalinyl substituted compounds **81**, obtained after condensation with **80**, showed an onset around 500–550 nm.

**Scheme 23:**
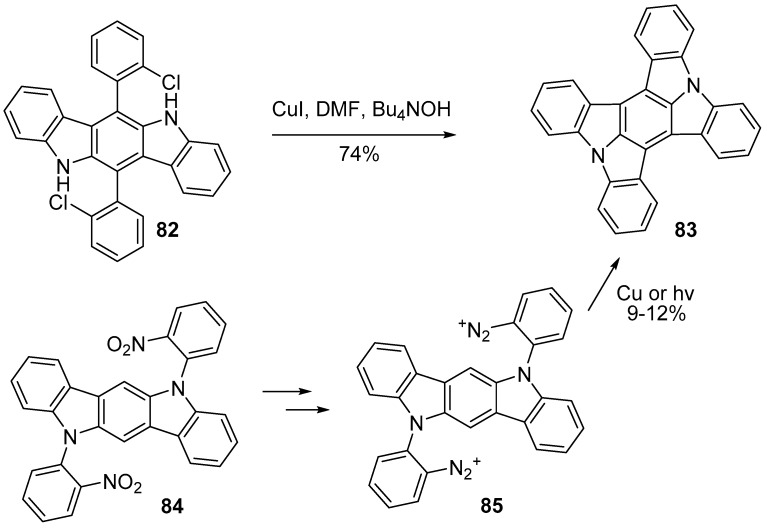
Synthesis of dibenzo[2,3:5,6]pyrrolizino[1,7-*bc*]indolo[1,2,3-*lm*]carbazole.

Khodorkovsky and coworkers prepared a new fused indolocarbazole donor system **83**, by two different approaches. The first is starting from 6,12-bis(2-chlorophenyl)-5,11-dihydroindolo[3,2-*b*]carbazole **82**, which twice undergoes an intramolecular Buchwald–Hartwig amination. The second method begins with 5,11-bis(2-nitrophenyl)-5,11-dihydroindolo[3,2-*b*]carbazole **84**, also obtained through Buchwald–Hartwig amination of the parent indolo[3,2-*b*]carbazole. Reduction, diazotation to **85** and insertion at the meso position gives the same ring closed product **83**. The yield of the compound via this approach is however lower than for the previous method due to formation of other isomers (Scheme 23) [60,61].

**Scheme 24:**
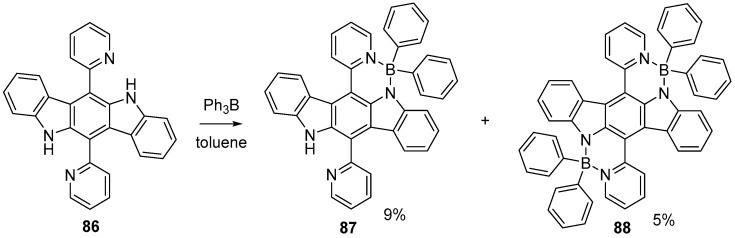
Complexation of indolo[3,2-*b*]carbazole with diphenylborane units.

Curiel *et al.* performed the condensation of indole with pyridine-2-carboxaldehyde to obtain indolo[3,2-*b*]carbazole **86**. This indolo[3,2-*b*]carbazole has then been converted into a complex with triphenylborane to obtain products **87** and **88** (Scheme 24). The maximum absorption peak in DCM shifted from 441 nm (no complexation), over 545 (once complexed) to 643 nm (double complexation) [62].

**Scheme 25:**
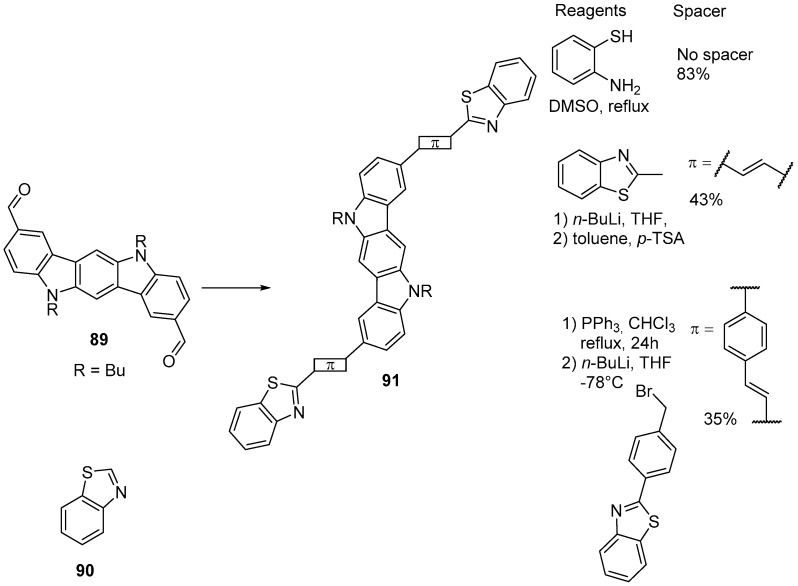
Donor–acceptor systems containing indolo[3,2-*b*]carbazole.

Shi *et al.* prepared a series of donor–acceptor systems starting from indolo[3,2-*b*]carbazole-2,8-dicarbaldehyde **89**. This compound was coupled with benzo[*d*]thiazole **90** to form **91**, either directly or using various π-spacers (Scheme 25). The compound without spacer showed an onset in the absorption spectrum at 425 nm and a single peak at 352 nm in different solvents. For the compounds with the spacers the onset was around 475 nm and peaks at 370–420 nm (low energy) and 300–370 nm (high energy) [63].

**Scheme 26:**
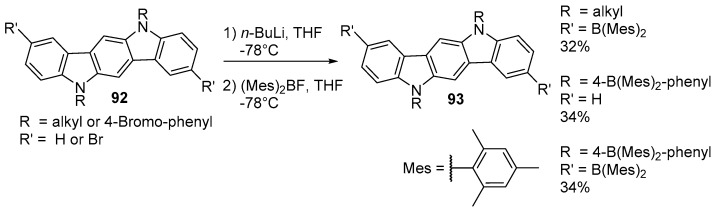
Introduction of mesityl groups on indolo[3,2-*b*]carbazole.

Shi *et al.* introduced dimesitylboron-groups at various positions of the indolo[3,2-*b*]carbazole **92** to obtain compounds **93** with an absorption onset around 410–420 nm (Scheme 26). These compounds show quite high fluorescence quantum yields (up to 0.76) [64,65]. Shi *et al.* also prepared a combination of the above-mentioned systems, *i.e.*, a benzothiazole moiety at one side and a dimesitylboron group at the other end of indolo[3,2-*b*]carbazole [66].

Ong and coworkers converted indolo[3,2-*b*]carbazoles to homopolymers. When they used *N*-alkylated parent indolo[3,2-*b*]carbazole **94** to undergo oxidative FeCl_3_-mediated polymerization, they obtained the “para-polymer” **95**. This means the indolo[3,2-*b*]carbazole is polymerized at the position para to the nitrogen atoms (2,8-positions).

They also performed a dehalogenative polymerisation on chlorinated indolo[3,2-*b*]carbazoles **96** and **97**. The 2,8-dichloro-indolo[3,2-*b*]carbazole **96** gave the “para-polymer” **95**; however the polydispersity index was lower with this method (1.16–1.20 instead of 2.08–2.63 for oxidative polymerization). The 3,9-dichloro-indolo[3,2-*b*]carbazole **97** on the other hand yielded the “meta-polymer” **98**.

**Scheme 27:**
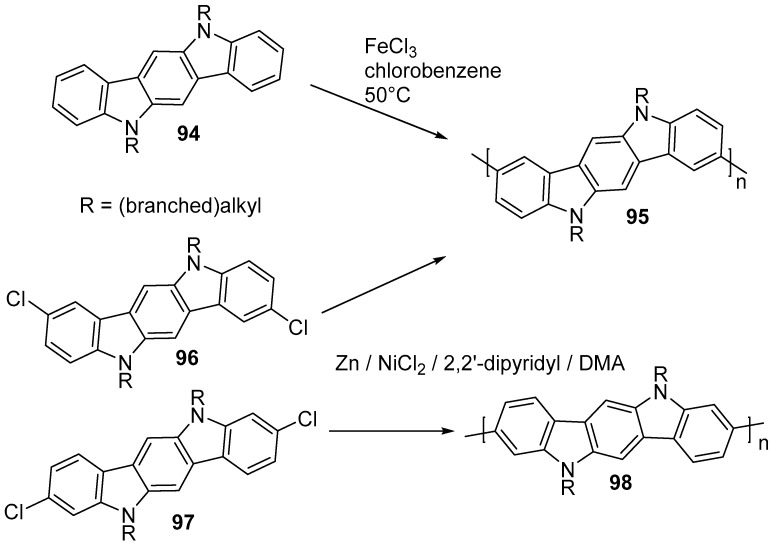
Polymerization of indolo[3,2-*b*]carbazole.

The absorption spectrum of the para-polymer was almost similar to the spectrum of the free indolo[3,2-*b*]carbazole (absorption onset at 370 nm in THF) . The meta-polymer on the other hand showed an onset of absorption in THF at 450 nm. This shift in absorption is ascribed to the π-conjugation along the indolo[3,2-*b*]carbazole backbone (Scheme 27) [67]. Leclerc *et al.* prepared polyindolo[3,2-*b*]carbazoles with and without bithiophene spacers [68,69] by performing palladium catalyzed couplings.

**Scheme 28:**
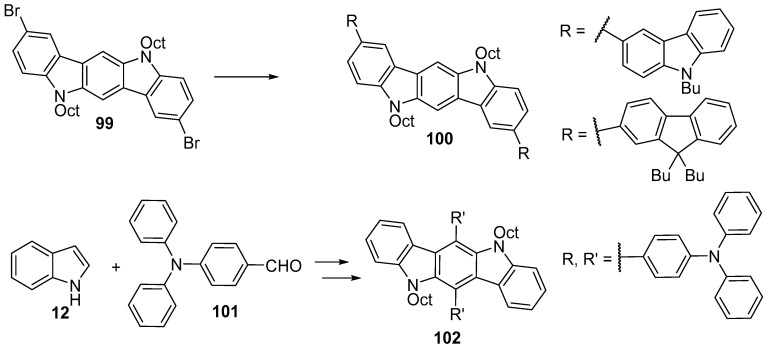
Functionalized indolo[3,2-*b*]carbazoles towards OLED’s.

Tao *et al.* prepared some indolo[3,2-*b*]carbazoles **100** and **102** with substitutions at the meso-positions, and at the 2,8-positions The former have been obtained by Suzuki coupling of brominated indolo[3,2-*b*]carbazole **99** with the different boronic acids to obtain **100** (Scheme 28).

The latter have been obtained by incorporating the substitutent in the starting aldehyde **101**, which is condensed with indole **12** to form indolo[3,2-*b*]carbazole **102**.

The absorption spectrum of these compounds showed peaks at 288–354 nm (dichloromethane solution) and 302–356 nm (in films). The compounds showed emission peaks (in dichloromethane) at 435–444 nm and at 436–450 nm (in films) [6,70]. Some other interesting similar structures of this kind were prepared by Leclerc *et al*. Here the indolo[3,2-*b*]carbazoles were end-capped with thiophene, benzene and styrene moieties [71]. Grazulevicius *et al.* expanded the scope of the reaction with various aromatic systems [72]. Liu *et al.* performed a double Heck reaction on dibromoindolo[3,2-*b*]carbazole to connect triphenylamine with the use of an alkene spacer [73].

**Scheme 29:**
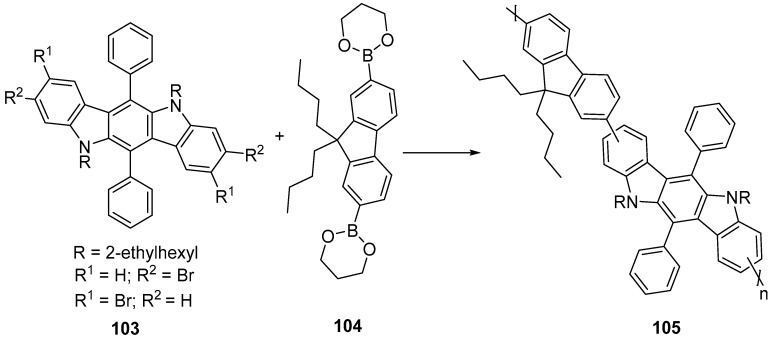
Copolymerization of indolo[3,2-*b*]carbazole with fluorene.

Chen *et al.* copolymerized both 2,8-dibromo-indolo[3,2-*b*]carbazole **103** and 3,9-dibromoindolo[3,2-*b*]carbazole **103** with 9,9-dibutyl-fluorene **104** to obtain copolymers **105** (Scheme 29) with absorption peaks (in THF) at 357 nm (2,8-isomer) and 392 nm (3,9-isomer). Photoluminescence was at 437 nm and 457 nm respectively (in THF) [74].

**Scheme 30:**
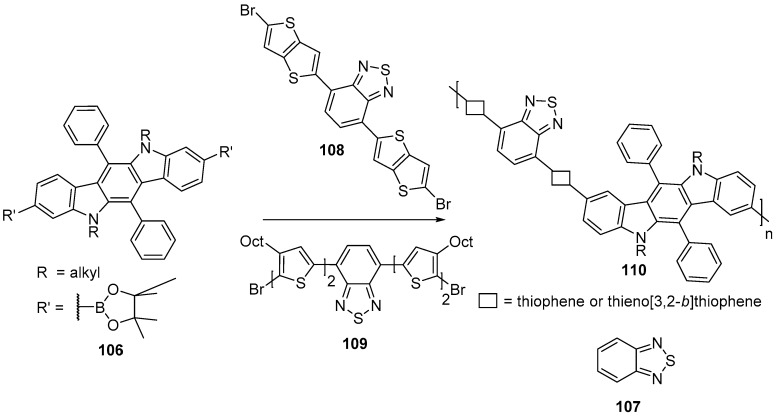
Donor–acceptor polymer with benzothiadiazole acceptor.

Fan *et al.* and Lu *et al.* both prepared copolymers consisting of indolo[3,2-*b*]carbazole **106** as the donor-system and benzothiadiazole **107** as the acceptor part. Fan used a thieno[3,2-*b*]thiophene spacer **108**, while Lu inserted an alkylated thiophene spacer **109** (Scheme 30). The polymer **110** with the thiophene spacers showed an absorption peak (in chloroform) at 538 nm and showed a weak absorption up to 650 nm. The one with the thieno[3,2-*b*]thiophene spacer showed two peaks, one at 420 nm and one at 570 nm (in dichlorobenzene solution and in thin film) and had absorption up to 675 nm [75,76]. For another example, see Hashimoto *et al.* [77].

**Scheme 31:**
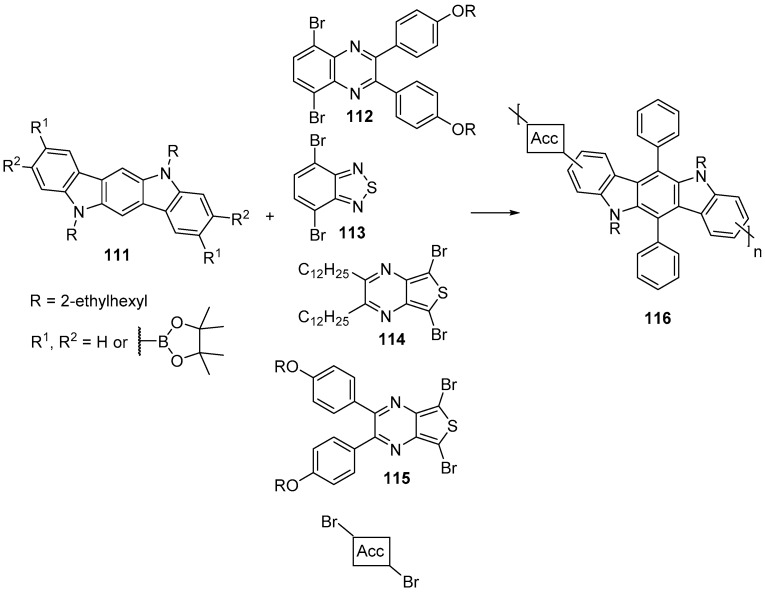
Donor–acceptor polymers with various acceptor parts.

Chen *et al.* prepared several donor–acceptor alternating copolymers **116** starting from 2,8- and 3,9-diboronate esters **111** as the indolo[3,2-*b*]carbazole donorsystem. These indolo[3,2-*b*]carbazoles are coupled with four different dibrominated acceptorsystems **112**, **113**, **114** and **115** by performing a Suzuki coupling (Scheme 31) [78].

**Scheme 32:**
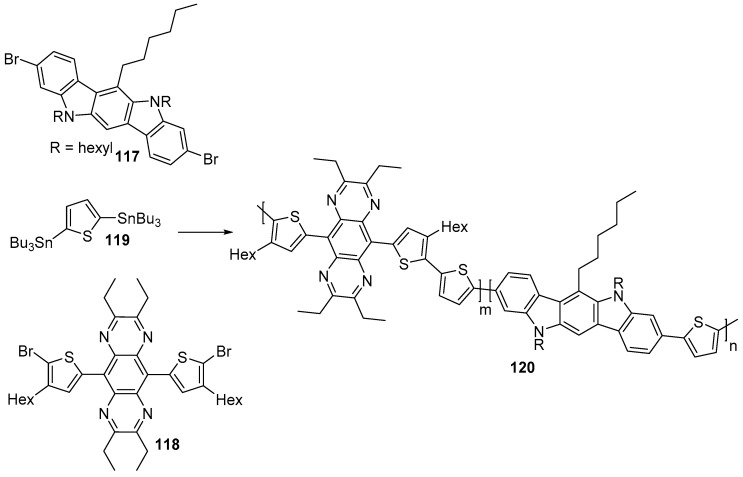
Copolymerization of indolo[3,2-*b*]carbazole with pyrazino[2,3-*g*]quinoxaline.

Peng *et al.* prepared copolymers of indolo[3,2-*b*]carbazole **117** with pyrazino[2,3-*g*]quinoxaline **118** by realizing a Stille coupling with the dibrominated compounds mentioned above and bis-(tributylstannyl)thiophene **119** (Scheme 32). The polymers **120** showed an absorption onset from 800 nm (in THF) [79].

**Scheme 33:**
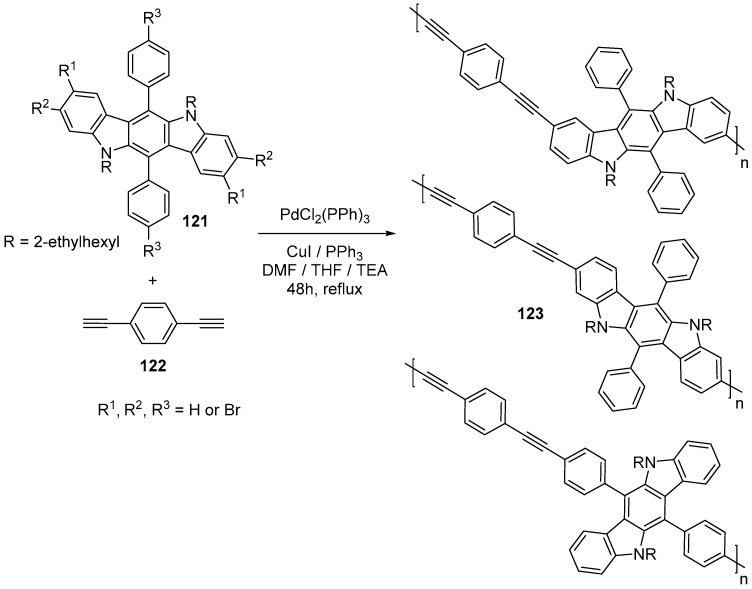
Polymerization of indolo[3,2-*b*]carbazole with π-spacer.

Grigoras *et al.* prepared polymers starting from brominated indolo[3,2-*b*]carbazoles **121** and 1,4-diethynylbenzene **122** by performing a Sonogashira coupling (Scheme 33). All polymers **123** show an absorption onset around 470 nm (in chloroform and thin film). The peaks of the absorption spectrum are located at 350 nm for the para- and the meso-polymer. The meta-polymer had a peak at 400 nm. We can again conclude that polymerization is best performed at the 3,9-positions and that the spacer used will cause a higher effective conjugation length [80].

**Scheme 34:**
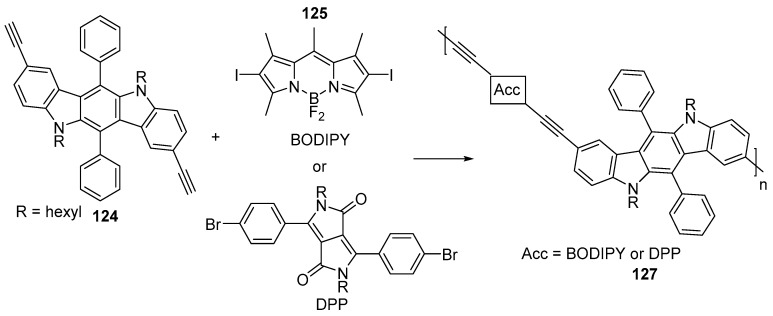
Donor–acceptor polymers.

Dehaen *et al.* prepared polymers **127** by performing Sonogashira couplings on 2,8-dialkynyl-indolo[3,2-*b*]carbazole **124** and halogenated acceptor systems like BODIPY **125** or DPP (diketopyrrolo[3,4-*c*]pyrrole) **126** (Scheme 34). The polymer with the DPP functionality shows a peak in the absorption spectrum at 505 nm and an onset around 600 nm. The polymer with the BODIPY core on the other hand has a peak at 536 nm and an onset around 700 nm (all in chloroform solution) [81]. Yagai *et al.* synthesized similar indolo[3,2-*b*]carbazoles, end-capped with a DPP functionality connected to the indolo[3,2-*b*]carbazole without an alkyn spacer. These molecules showed an onset in the absorption spectrum at 650 nm (in chloroform) [82].

## 4. Smaller Organic Donor Systems

### 4.1. Pyrrolo[2,3-f]indole

**Scheme 35:**
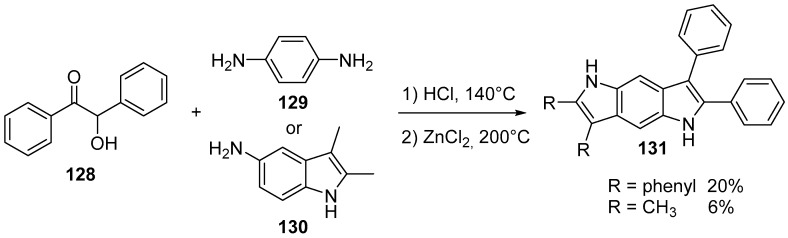
Synthesis of pyrrolo[2,3-*f*]indole.

The earliest synthesis of pyrrolo[2,3-*f*]indole was reported by Kingsley and Plant by condensing benzoin **128** with either 1,4-phenylenediamine **129** or 5-amino-indole **130** in a two-step procedure (Scheme 35). The synthesis starting from 1,4-phenylenediamine **129** gave the desired compound **131** in 20% yield. Starting from 5-aminoindole **130**, the yield was only 6%.

The angular isomer (pyrrolo[3,2-*e*]indole) was also formed during the reaction, the yield however was even lower for this compound. They showed an onset in the absorption spectrum at lower wavelengths, which makes the linear systems (pyrrolo[2,3-*f*]indole) more interesting for long wavelength absorption [12].

**Scheme 36:**
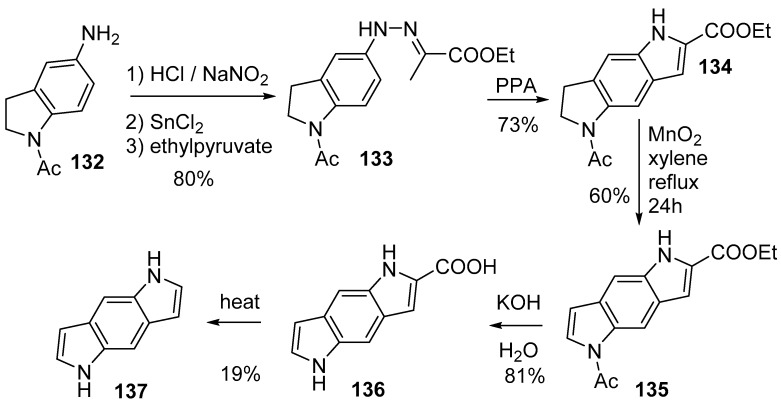
Alternative synthesis of pyrrolo[2,3-*f*]indole.

Samsoniya *et al.* reported the synthesis of the parent pyrrolo[2,3-*f*]indole **137** in 1977, starting from 5-amino-indoline **132** which first undergoes diazo coupling, reduction and condensation with ethylpyruvate to form intermediate **133**. Fischer indolization in acidic medium affords tetrahydropyrrolo[2,3-*f*]indole **134**, followed by oxidation to afford pyrrolo[2,3-*f*]indole **135**. Deprotection and saponification of **135** gives compound **136**, which is decarboxylated to the parent pyrrolo[2,3-*f*]indole **137** (Scheme 36) [83,84].

**Scheme 37:**
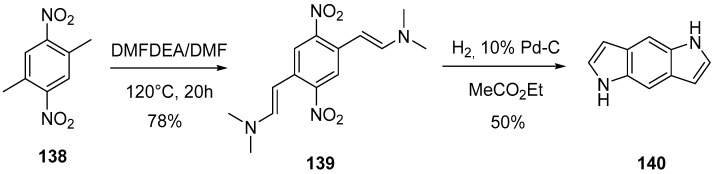
Berlin pyrrolo[2,3-*f*]indole synthesis.

Berlin *et al.* prepared pyrrolo[2,3-*f*]indoles and other isomers from dinitroxylenes in a two-step procedure. Condensation of the methyl group of dinitroxylene **138** with dimethylformamide diethylacetal and successive reduction of the obtained compound **139** gave pyrrolo[2,3-*f*]indole **140** in 40% overall yield (Scheme 37) [85].

**Scheme 38:**
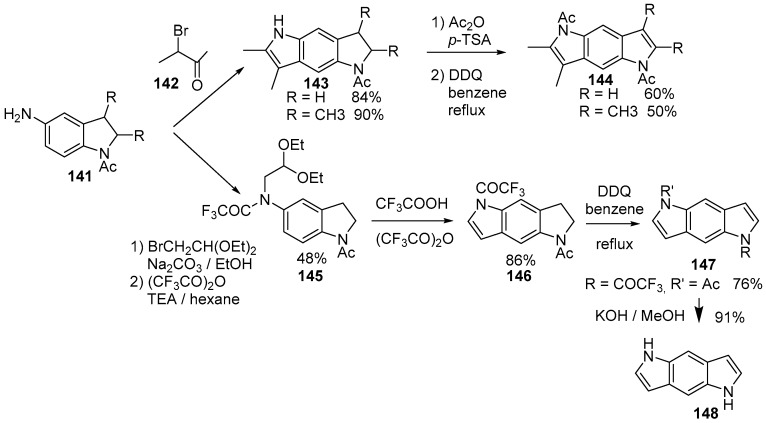
Condensation reactions of 5-amino-indoline.

Dmitrienko *et al.* condensed 5-amino-indoline **141** with 3-bromo-2-butanone **142** to get the linear substituted tetrahydropyrrolo[2,3-*f*]indole **143**, which is oxidized with DDQ to pyrrolo[2,3-*f*]indole **144** (42% overall), whereas the angular isomer was obtained when using 5-amino-indole. The same researchers also demonstrated that it was possible to get the unsubstituted pyrrolo[2,3-*f*]indole **148** in a multistep procedure by condensing indoline **141** with bromoacetaldehyde diethyl acetal to form **145**. This compound was ring closed to **146**, oxidized to **147** and deprotected to **148** in 25% overall yield (Scheme 38) [86].

**Scheme 39:**
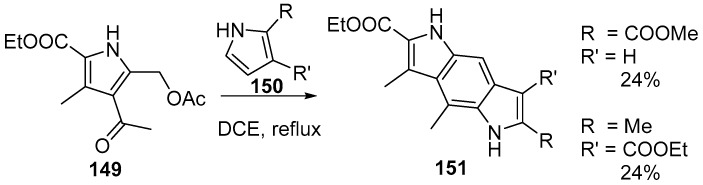
Condensation reaction of pyrroles to form pyrrolo[2,3-*f*]indoles.

Chunchatprasert *et al.* have prepared pyrrolo[2,3-*f*]indole **151** (24%) by condensing biselectrophilic 5-acetoxy-4-acetylpyrrole **149** with 2,3-unsubstituted pyrrole **150** under the influence of montmorillonite clay in 1,2-dichloroethane solvent (Scheme 39) [87].

**Scheme 40:**
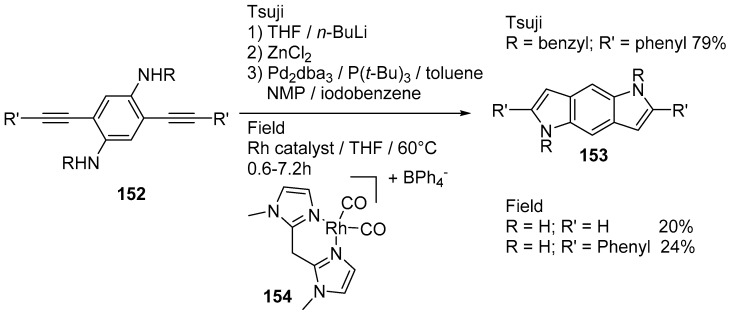
Pyrrolo[2,3-*f*]indole synthesis starting from 1,4-di-amino-2,5-di-alkynylbenzene.

Both Field *et al.* and Tsuji *et al.* have prepared pyrrolo[2,3-*f*]indoles from 2,5-dialkynyl-1,4-phenylenediamine **152** via transition metal catalyzed reactions. Tsuji used phenyl substituted alkynes and benzyl substituted amines to afford the pyrrolo[2,3-*f*]indole **153** in 79% yield [88]. Field however employed substituted alkynes and unprotected amine groups on the starting material to get around 20% yield of the non-substituted pyrrolo[2,3-*f*]indole **153** by using a rhodium catalyst **154** (Scheme 40) [89].

In 2011, Miura *et al.* improved the method of Field by carrying out a one pot double cyclisation-*N*-arylation on **155** to obtain pyrrolo[2,3-*f*]indole **156** in 43% yield [90].

**Scheme 41:**
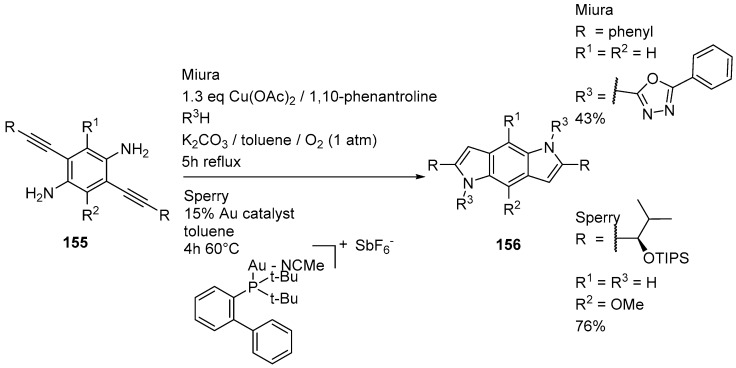
Improved synthesis of pyrrolo[2,3-*f*]indole starting from 2,4-dialkynyl-1,3-diaminobenzene.

Sperry *et al.* have shown that it was possible to further improve the reaction by using a gold catalyst **157** to realize the double cyclisation starting from free amino groups and substituted alkynes on starting material **155** in 76% yield (Scheme 41) [91].

**Scheme 42:**
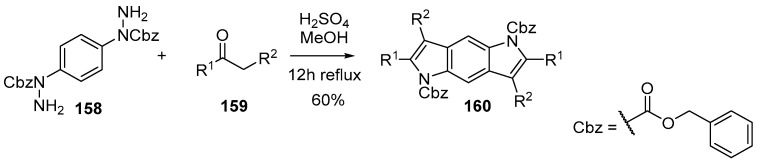
Double Fischer indolization to obtain pyrrolo[2,3-*f*]indole.

Cho *et al.* started their synthesis from 1,4-phenylene bishydrazide **158** to perform a double Fischer indolization using various ketones **159**. The linear pyrrolo[2,3-*f*]indole **160** was the major product of this reaction (up to 60%), accompanied by the angular by-product which is not shown here (Scheme 42) [92].

**Scheme 43:**
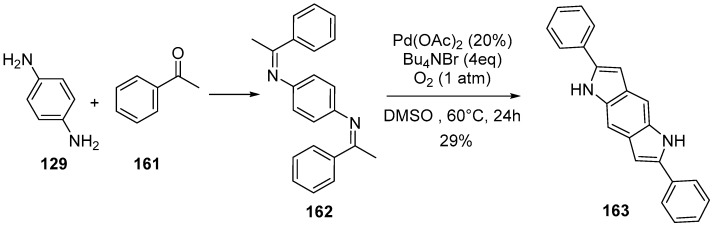
Oxidative indolization starting from aniline and ketones.

Yoshikai *et al.* optimized the reaction of anilines with ketones to oxidatively form indoles via an *N*-aryl imine intermediate. These optimized conditions were used to perform a double indole formation starting from 1,4-phenylenediamine **129** and acetophenone **161**. Pyrrolo[2,3-*f*]indole **163** is formed in 29% yield via intermediate **162** (Scheme 43) [93]. 

**Scheme 44:**
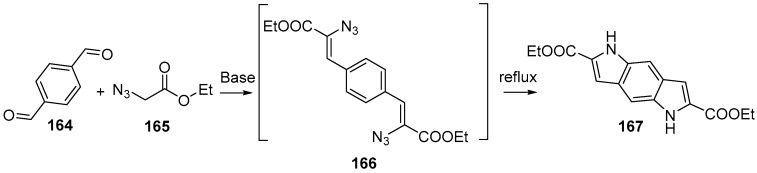
Hemetsberger double indole synthesis.

Liotta *et al.* prepared the pyrrolo[2,3-*f*]indole scaffold starting from terephthaldehyde **164**. This compound is condensed with two equivalents of ethyl-2-azidoacetate **165**, after which the obtained compound **166** is thermally closed by nitrene insertion to obtain the final pyrrolo[2,3-*f*]indole **167** in 72% yield (Scheme 44) [94].

**Scheme 45:**
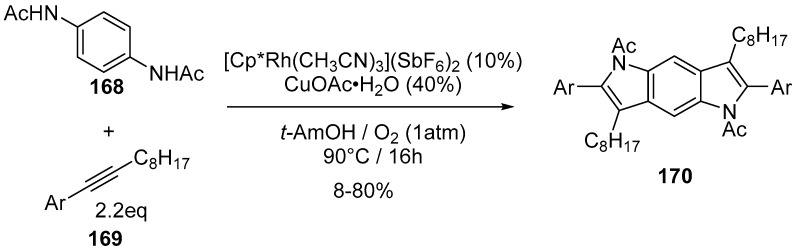
Pyrrolo[2,3-*f*]indole synthesis starting from *N*,*N*′-(1,4-phenylene)diacetamide.

Tokoro *et al.* developed a transition metal-catalyzed C-H activation to convert *N,N′*-(1,4-phenylene)diacetamide **168** and an arylalkyne **169** into substituted pyrrolo[2,3-*f*]indole **170** (Scheme 45). The reaction works well with simple aryls (8%–63%) as well with electron deficient systems (benzothiadiazole (76%), fluorenone (80%)) and electron rich systems (carbazole (64%)) [95].

### 4.2. Pyrrolo[3,2-b]carbazole

**Scheme 46:**
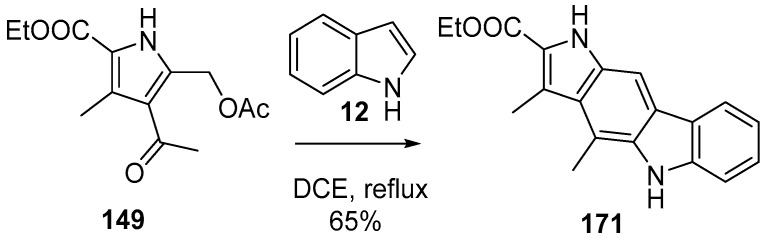
Condensation to form pyrrolo[3,2-*b*]carbazole.

Chunchatprasert *et al.* used the method as described earlier to prepare pyrrolo[3,2-*b*]carbazoles starting from the same biselectrophilic pyrrole **149**. In this case, 2,3-unsubstituted indole **12** was used to obtain pyrrolo[3,2-*b*]carbazole **171** in 65% yield (Scheme 46) [96]. 

**Scheme 47:**
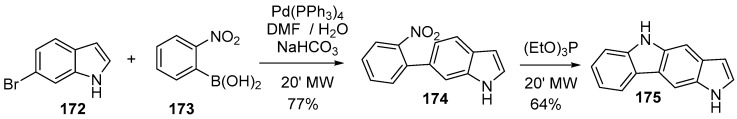
Microwave assisted Cadogan reaction.

Van der Eycken *et al.* performed a microwave assisted Cadogan ring closure on the Suzuki coupled product **174** of 6-bromo-indole **172** and 2-nitro-phenylboronic acid **173**, using triethyl phosphite to obtain pyrrolo[3,2-*b*]carbazole **175** (Scheme 47) [97].

Nagarajan *et al.* used 3-amino-carbazoles **176**, ethylene glycol **177** and a ruthenium catalyst to prepare pyrrolo[2,3-*c*]carbazoles **179** in good yield (73%), however this affords the angular isomer instead of the linear [98].

When Nagarajan *et al.* applied their procedure, using 3-amino-carbazoles **176** and propargyl alcohol **178**, catalyzed by Zn(OTf)_2_, they again obtained the angular isomer, pyrrolo[2,3-*c*]carbazole **179** (75%) instead of the linear isomer [99].

**Scheme 48:**
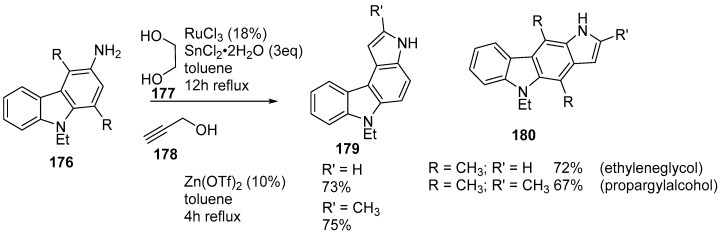
Pyrrolocarbazole synthesis.

By blocking the 4-position with a methyl group, both methods were suitable to prepare the linear isomer instead of the angular one that is normally formed. The yield of pyrrolo[2,3-*b*]carbazole **180** formation is 72% and 67%, respectively (Scheme 48).

**Scheme 49:**
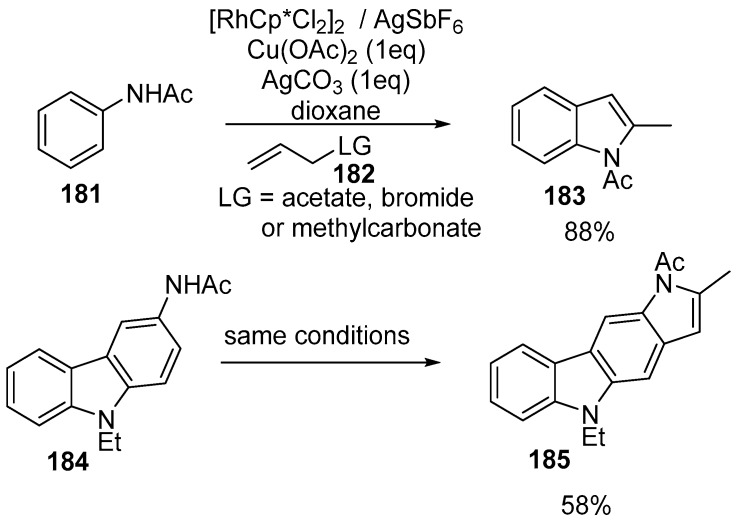
Rh-catalyzed pyrrolo[3,2-*b*]carbazole synthesis.

Fu *et al.* used *N*-aryl acetamide **181** and allyl acetate or other allyl moieties **182** to get indole **183** by using a rhodium catalyst. Yields for this synthesis were up to 88%. By using the optimized conditions on acetylated 3-amino-carbazole **184**, pyrrolo[2,3-*b*]carbazole **185** was obtained in 58% yield (Scheme 49) [100].

## 5. Heterocyclic Analogs

**Scheme 50:**
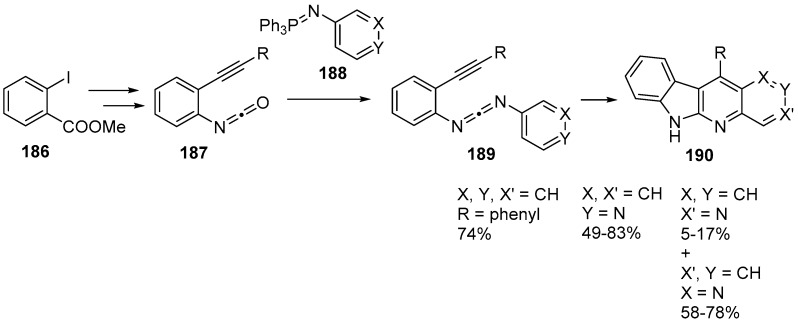
Synthesis of indolo[2,3-*b*]naphthyridines.

Wang *et al.* prepared several tetracyclic indolonaphthyridines **190** starting from methyl 2-iodobenzoate **186**. The first step is a Sonogashira coupling (88%–99%), followed by a saponification of the ester to get the corresponding carboxylic acid (61%–94%). Then a Curtius rearrangement is performed (73%–78%) and the obtained isocyanate **187** is subjected to an aza-Wittig reaction with **188**, immediately followed by thermal ring closure of the carbodiimide intermediate **189** to form the final product **190** (Scheme 50). When a pyridine analog of **188** is used, multiple isomers are possible [101].

**Scheme 51:**
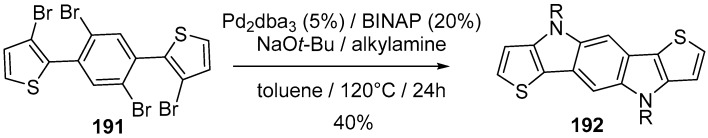
Synthesis of thiophene analog of indolo[3,2-*b*]carbazole.

Donaghey *et al.* obtained a heterocyclic analog of indolo[3,2-*b*]carbazole where the outer benzo rings are replaced by thieno rings. The reaction starts from tetrabrominated 2,2′-(2,5-dibromo-1,4-phenylene)-bis-(3-bromothiophene) **191**, which undergoes a quadruple Buchwald-Hartwig amination to obtain the pentacyclic pyrroloindacenodithiophene (thieno[2′,3′:4,5]pyrrolo[2,3-*f*]thieno[3,2-*b*]indole) **192** (40%) (Scheme 51) [102]. This building block will be used further on in the section about polymers and properties (*vide intra*).

**Scheme 52:**
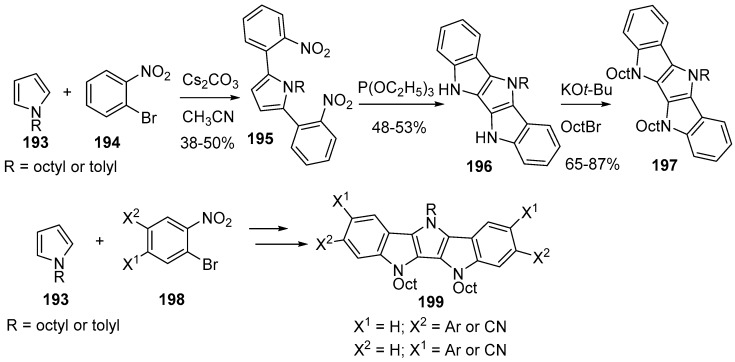
Pyrrolo[3,2-*b*:4,5-*b*′]diindole synthesis.

Mo *et al.* synthesized an indolo[3,2-*b*]carbazole analog in which the middle aromatic ring is replaced by a pyrrole ring to form a pyrrolo[3,2-*b*:4,5-*b*′]diindole **197**. The synthesis starts from *N*-alkylated or *N*-arylated pyrrole **193** that is coupled with two molecules of 2-bromo-nitrobenzene **194** to form **195** in moderate yield (38%–50%). Then, a double Cadogan ring closure (48%–53%) is performed to obtain **196**, which is alkylated twice in 65%–87% yield to obtain the final compound **197** (Scheme 52) [103].

The reaction is also possible starting from 2,4-dibromo-nitrobenzene or 2,5-dibromo-nitrobenzene **198** (38%–61%) to obtain dibrominated compounds **199**. These compounds are further functionalized by Suzuki, Stille and Yamamoto coupling. The non-functionalized compound shows an absorption onset at 385–390 nm. The compounds with functionalization at the 2- and 9-position (X^1^ = Ar or CN) do not show different properties. The 3,8-functionalized isomer (X^2^ = Ar or CN) show a slight red-shift in the absorption spectrum (30–50 nm).

## 6. Larger Systems

**Scheme 53:**
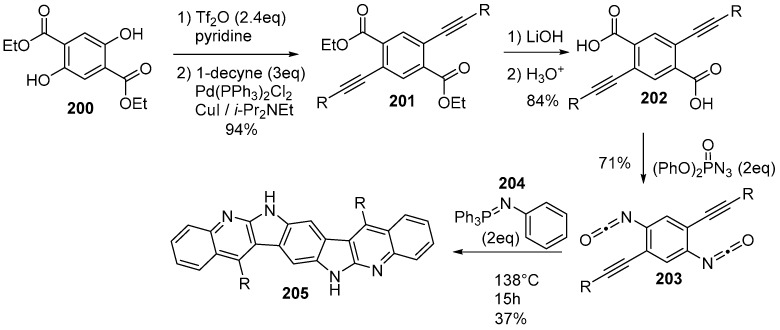
Synthesis of heptacyclic system.

Earlier we mentioned a method to prepare indolonaphthyridines from methyl 2-iodo-benzoate (Scheme 50) [67]. The authors started from diethyl 2,5-dihydroxyterephthalate **200** to quantitatively convert this compound to diethyl 2,5-dialkynylterephthalate **201** in two steps, which now was used as a substrate for a double cyclization reaction. After saponification to **202**, Curtius rearrangement to **203**, aza-Wittig reaction with **204** and heating for 15 h, a heptacyclic polyheteroaromatic system **205** was obtained. The final step of the reaction however only worked with phenyl substituted iminophosphoranes **204** and not with the pyridine analogs (Scheme 53) [101].

**Scheme 54:**
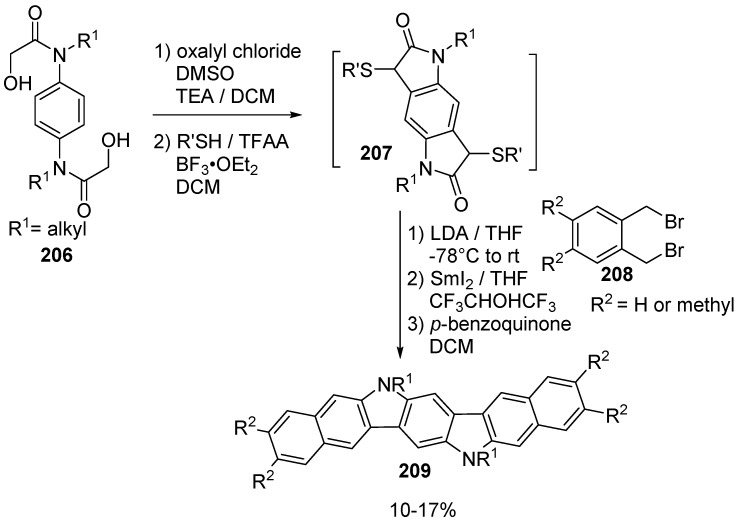
Dibenzoindolo[3,2-*b*]carbazole synthesis.

Turner *et al.* prepared dibenzoindolo[3,2-*b*]carbazoles **209** in 10%–17% overall yield using a multistep procedure involving the condensation of pyrroloindole intermediate **207** and ortho-xylylene derivative **208**. The intermediate **207** was prepared from a 1,4-disubstituted benzenebisamide **206** (Scheme 54) [104].

**Scheme 55:**
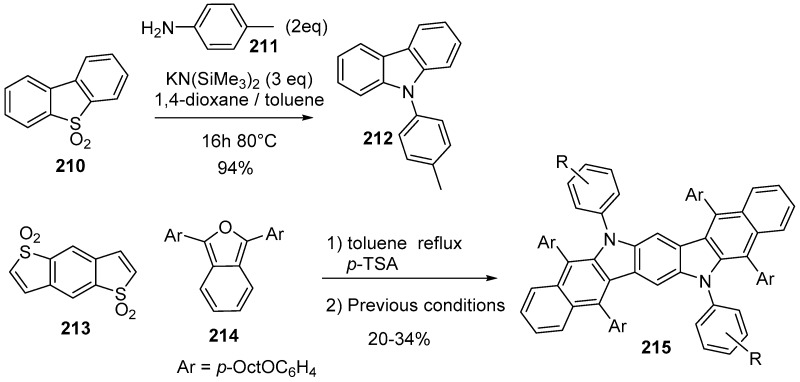
Dibenzoindolo[3,2-*b*]carbazole synthesis starting from benzodithiophenedisulfone.

Yorimitsu *et al.* started from dibenzothiophene, which was oxidized to sulfone **210** by using aqueous hydrogen peroxide. In the next step, an aniline **211** is used to perform a nucleophilic aromatic substitution to obtain the corresponding carbazole **212** in 94% yield (Scheme 55).

On yet another substrate, benzothiophenesulfone, the authors first performed a Diels–Alder reaction with isobenzofuran **214** to afford the expanded benzonaphthothiophene sulfone, which can be converted to benzo[*b*]carbazole (not shown, 62%).

When using the bifunctional benzodithiophenedisulfone **213**, and applying the same methodology, dibenzoindolo[3,2-*b*]carbazole **215** can be obtained in 22%–38% overall yield (Scheme 55) [105]. 

Sung *et al.* performed a double Fischer indolization on 1,4-phenylene bishydrazide **147** and 3,4-dihydronaphthalen-1(2*H*)-one to obtain another isomer of dibenzoindolo[3,2-*b*]carbazole [106] (not shown).

**Scheme 56:**
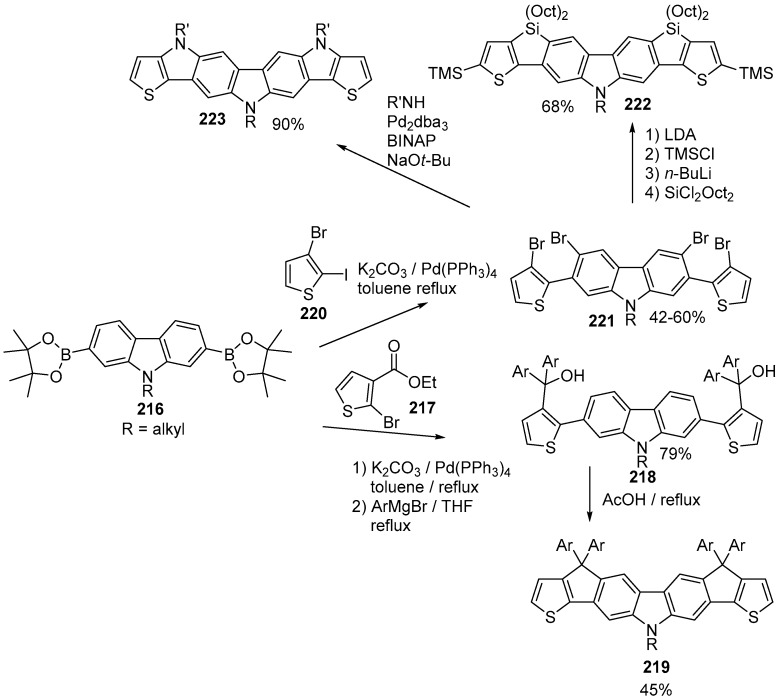
Synthesis of heptacyclic carbazole analogs.

Hsu *et al.* prepared three heptacyclic carbazole derivatives **219**, **222** and **223** by different annelation reactions to a carbazole precursor **216**. The first one contains an sp^3^ center between the carbazole moieties and the two thiophene rings. The two thiophene rings **217** were linked by a Suzuki reaction with carbazole **216**, followed by Grignard addition of four aryl groups to the two ester groups to obtain **218**. Acid catalyzed ring closure of the intermediate biscarbinol gave the final heptacyclic compound **219** (Scheme 56) [107].

For the two other analogs, the ester functionality in the thiophene starting material **220** was replaced by a bromine atom and additionally carbazole was dibrominated after protection of the α-positions of thiophene to get **221**.

To obtain the bis(silacyclopentadiene) compound, the four bromine atoms are lithiated and the end product **222** is formed by addition of SiCl_2_Oct_2_ (94%).

The pyrrole analog did not require protection of thiophene. The tetrabrominated product was subjected to Buchwald–Hartwig amination to obtain the heptacyclic pyrrole analog **223** (90%) (Scheme 56) [108]. 

## 7. Polymerization and Applications 

**Scheme 57:**
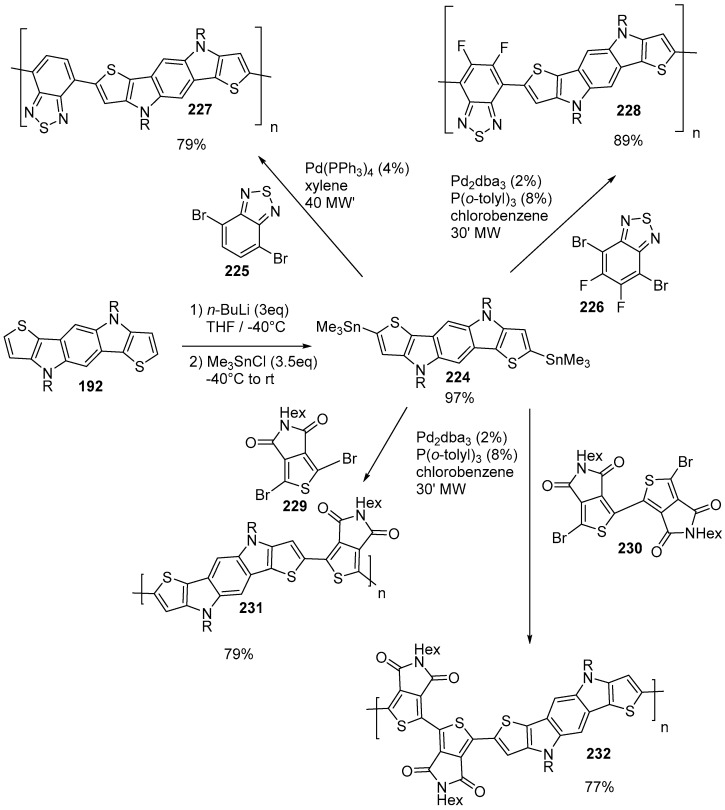
Polymer synthesis with heterocyclic indolo[3,2-*b*]carbazole analog.

The pyrroloindacenodithiophenes **192** (thieno[2′,3′:4,5]pyrrolo[2,3-*f*]thieno[3,2-*b*]indole) prepared by Donaghey *et al.* (Scheme 51) were connected with different acceptors by Stille coupling with the stannylated compound **224** to obtain an alternating copolymer (Scheme 57). When using benzothiadiazole **225** or difluorbenzothiadiazole **226**, absorption spectra (in chloroform) showed peaks up to 900 and 850 nm for polymers **227** and **228**, respectively.

The authors also used two different acceptors to copolymerize with their donor systems: 1,3-dibromo-5-octylthieno[3,4-*c*]pyrrole-4,6-dione **229** and 3,3′-dibromo-5,5′-di-2-ethylhexyl 1,1’-bi(thieno[3,4-*c*]pyrrole)-4,4’,6,6’(5H,5′H)-tetrone **230**.

These polymers (**231** and **232** respectively) showed less red shifted absorption (up to 750 nm) in comparison to the two previous systems [102].

**Scheme 58:**
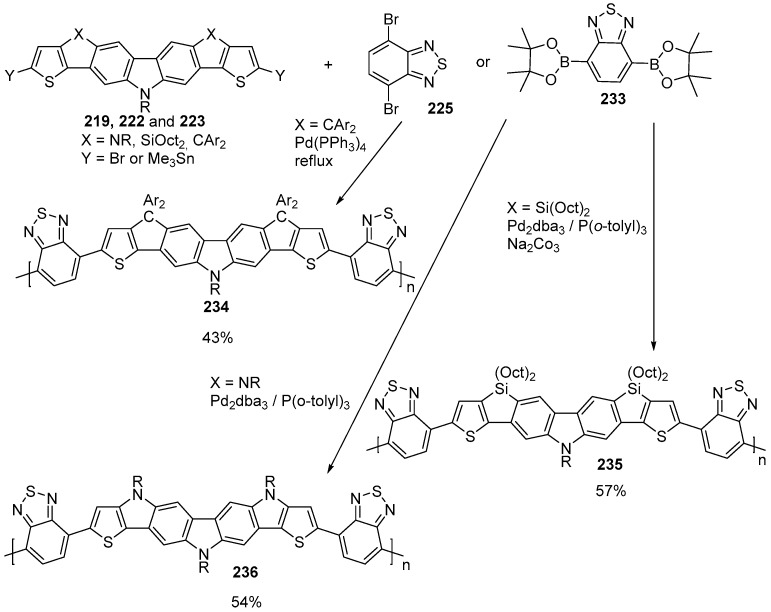
Polymer synthesis with heptacyclic ring systems.

The silicon **222**, carbon **219** and nitrogen **223** bridged heptacyclic systems described by Hsu (Scheme 56) were co-polymerized with benzothiadiazole **225** or **233** (Scheme 58). An alternating co-polymer was obtained after Stille or Suzuki coupling [107,108].

While the monomers show absorption up to 400 and 460 nm in toluene, the polymers show absorption up to 700 nm (carbon **234** and silicon **235** bridge) and 840 nm (nitrogen **236** bridge) [108].

**Scheme 59:**
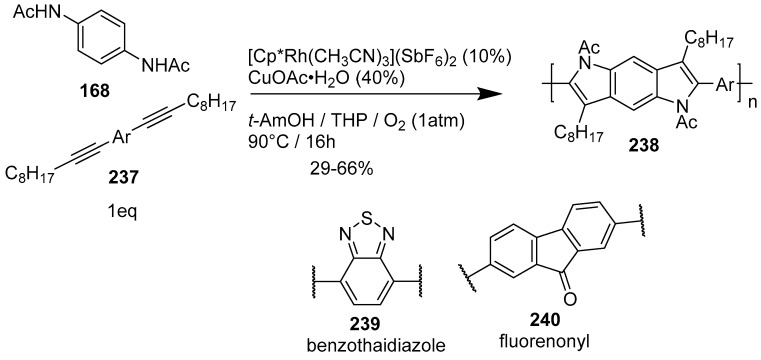
Synthesis of alternating polymer containing pyrrolo[2,3-*f*]indole core.

Tokoro *et al.* prepared interesting alternating co-polymers **238** with pyrrolo[2,3-*f*]indole and aryl building blocks. The one-step reaction starts from *N*,*N*′-diacetyl-*p*-phenylenediamine **168** and 1,4-dialkynyl-benzene **237** (Scheme 59). It was also proven that the benzene core of the di-alkynyl could be replaced by either electron-rich as electron-poor aryls to obtain various donor–acceptor systems. The absorption spectrum of the polymer with a benzene moiety in the backbone showed a maximum at 365 nm (in DCM solution). When electron withdrawing systems like benzothiadiazole **239** or fluorenonyl **240** were used instead of benzene, the absorption peak shifted to 442–453 nm [95].

## 8. Conclusions

The past two decades have seen a large activity in the domain of indolo[3,2-*b*]carbazoles and the related smaller and larger benzodipyrrole analogs. New synthetic methods were reported, leading to superior materials. Certainly, the full potential of this work has not been realized. It is expected that we will see further development in this area.

## Abbreviations

The following abbreviations are used in this manuscript:

Ac	Acetyl
Ac_2_O	Acetic anhydride
AcOH	Acetic acid
BHT	3,5-dibutyl-4-hydroxytoluene
BINAP	2,2′-bis(diphenylphosphino)-1,1′-binaphtyl
Bz	Benzoyl
Cbz	Carboxybenzyl
dba	dibenzylideneacetone
DCE	1,2-dichloroethane
DCM	dichloromethane
DDQ	2,3-dichloro-5,6-dicyano-1,4-benquinone
DMA	Dimethylacetamide
DMF	Dimethylformamide
DMFDEA	Dimethylformamidediethylacetal
DMSO	Dimethylsulphoxide
DPP	Diketopyrrolo[3,4-*c*]pyrrole
DSSC	Dye sensitized solar cell
EtOH	Ethanol
HMPA	Hexamethylphosphoramide
LDA	Lithium diisopropylamide
MeOH	Methanol
NBS	*N*-bromo-succinimide
Nf	Nonaflyl
NMP	*N*-methyl-2-pyrrolidone
OFET	Organic field effect transistor
OLED	Organic light emitting diode
OPV	Organic photovoltaic
Ph	Phenyl
PPA	Polyphosphoric acid
PPSE	Polyphosphoric acid trimethylsilyl ester
*p*-TSA	Para toluenesulphonic acid
*t*-AmOH	tertiary amylalcohol
TEA	Triethylamine
TFAA	Trifluoroacetic anhydride
Tf	Triflate
THF	Tetrahydrofuran
THP	Tetrahydropyran
TIPS	Triisopropylsilyl
TMSCl	Trimethylsilylchloride
Ts	Tosyl
